# Effects of localization of uterine adenomyosis on clinical features and pregnancy outcome

**DOI:** 10.1038/s41598-023-40816-z

**Published:** 2023-09-07

**Authors:** Jinghua Shi, Yushi Wu, Xiaoyan Li, Zhiyue Gu, Chenyu Zhang, Hailan Yan, Yi Dai, Jinhua Leng

**Affiliations:** Department of Obstetrics and Gynecology, Peking Union Medical College Hospital, Peking Union Medical College and Chinese Academy of Medical Science, National Clinical Research Center for Obstetric and Gynecologic Diseases, Shuaifuyuan No. 1, Dongcheng District, Beijing, China

**Keywords:** Reproductive disorders, Outcomes research

## Abstract

The purpose of this study was to implore the association among clinical features, long-term fertility outcomes and the anatomical location of adenomyosis identified by ultrasound. We collected data of non-pregnant patients between 20 and 40 years old who had undergone surgical exploration for benign gynecological conditions at our institution between January 2010 and December 2017. A total of 158 women met the inclusion criteria and were allocated into three groups according to the ultrasound-determined adenomyosis anatomical location: anterior (Group A), posterior (Group B), both posterior and anterior (Group C). 44.3% (70/158) adenomyosis was located at the posterior side. History of miscarriage and parity were significantly higher in Group C (*p* = 0.036 and 0.001 respectively). Group C also had a higher concurrence rate of ovarian endometrioma (OEM) (80.4%, *p* = 0.002), pelvic adhesion (80.4%, *P* = 0.003) and the revised American Fertility Society (rAFS) Score (median64, range2-100, *P* < 0.001), while a significantly lower rate of concurrent peritoneal endometriosis (*P* = 0.01). Group B showed a relative higher rate of coexistent heavy menstrual bleeding (28.6%, *p* = 0.04) and oviduct obstruction (24.3%, *P* = 0.038). Group A had a higher proportion of coexistent leiomyoma (53.1%, *P* = 0.002). There were no significant differences between group A, B, and C in terms of pain symptoms, endometrial polyps, operation time, and endometriosis fertility index score and other basic characters (*p* > 0.05). During the follow-up, 59.2% (61/103) patients had clinical pregnancies, and 26.2% (16/61) of them experienced pregnancy loss. Total in vitro fertilization and embryo transfer pregnancy rate was 64.6% (42/65) and spontaneous pregnancy rate was 50.0% (19/38). The Kaplan–Meier curves demonstrated significant lower cumulative pregnancy rate in Group C than Group A and Group B (*p* = 0.01). Severe obstetric complications such as placenta previa, placenta accreta, preeclampsia, and preterm birth were only found in women with adenomyosis located in the posterior side. In conclusion, types of adenomyosis based on sonographic location had different clinical features and pregnancy outcome. Patients with adenomyosis lesion in both anterior and posterior sides had higher combination of OEM, pelvic adhesion and rAFS score.

## Introduction

Adenomyosis (AM) is a common benign gynecologic disorder that affects 8.8–61.5% of women undergoing a hysterectomy and 20–34% of women referred for pelvic imaging^1^. It is characterized by the presence of ectopic endometrial glands or stroma in the uterine myometrium^2^. Traditionally, the diagnosis of AM has been made histologically on the hysterectomy sample. However, surgery is restricted to the more severe symptomatic cases and therefore cannot be used as a classification tool for clinical use. Recent advances in imaging have made it possible to identify the disease in women who do not require or want a hysterectomy. Recent research has shown that ultrasonography (US) and Magnetic resonance imaging (MRI) have similar high sensitivity (0.81 vs. 0.71) and specificity (0.87 vs. 0.91) and are rarely both needed to make a diagnosis^3^. Moreover, ultrasonography is widely available and relatively inexpensive in office settings and relatively accurate when carried out by experts, making it the first-line imaging technique in gynecology.

There is a lack of an international consensus on an adenomyosis classification system^4^ that is useful for clinical practice and research. For a long time, researchers mainly focused on the diagnosis and clinical phenotype of “diffuse uterine adenomyosis” and “focal adenomyosis”. However, the localization of the disease has recently become part of the basis for categorization. Chapron et al.^5^ found that external adenomyosis was associated with deep infiltrating endometriosis (DIE) and was more common in young and nulliparous women whereas internal adenomyosis was more often associated with heavy menstrual bleeding. According to the sonographic reporting system for adenomyosis developed by Thierry Van den Bosch et al.^6^, the location of adenomyosis should be described as being anterior, posterior, lateral left, lateral right, or fundal. A new classification proposal^7^ published in 2020 also integrated the anatomical location (anterior, posterior, left lateral, right lateral, or fundal) into its classification system. However, whether this anatomical location affects the clinical features of adenomyosis and pregnancy prognosis has yet to be determined. The aim of this study was to compare the clinical presentation and pregnancy outcome of women affected with adenomyosis who had undergone surgery for benign gynecological conditions according to the ultrasound-diagnosed location of their adenomyosis.

## Materials and methods

### Study design, population, and data collection

We performed a retrospective cohort study to analyze data obtained from the medical records of 158 patients with adenomyosis from our hospital between January 2010 and December 2017. All patients had undergone fertility-sparing laparoscopic surgery and had the desire to conceive. All surgeries were performed according to relevant guidelines and regulations. Signed informed written consent was obtained from all of the included patients. The study protocol was approved by the ethics committee of our institution (the Institutional Review Board of our hospital, No. S-K1055). All of the data were fully anonymized before use.

The indications for surgery were a benign gynecologic disease that was associated with one of the symptoms below: (1) moderate to severe pain symptoms (e.g., dysmenorrhea, chronic pelvic pain, dyspareunia); (2) infertility which is defined as the inability to conceive despite frequent, unprotected sex for at least one year^8^; or (3) persistent pelvic masses (benign ovarian cysts, etc.). The exclusion criteria were as follows: (1)patients with cancer, (2) patitens with infectious disease, (3)patients who were currently pregnant, (4) patients who were younger than 20 years old or older than 40 years old During the surgery, endometriomas were removed, and peritoneal endometrial tissue was treated using bipolar electrocoagulation. Deep infiltrating endometriotic nodules and adenomyoma lesions were resected after consultation between the doctors and the patient, especially for those who had severe symptoms or repeated in vitro fertilization and embryo transfer (IVF-ET) failures. Adhesions were separated, uterine fibroids that may affect pregnancy and endometrial polyps were resected,and tuboplasty was performed for oviduct obstruction in infertile patients.

For each patient, socio-demographic and clinical data were obtained during face-to-face interviews conducted by the surgeon preceding the surgery at the outpatient department. The intensity of each patient’s pain symptoms related to dysmenorrhea, dyspareunia, and chronic pelvic pain (CPP) was evaluated using a 10cm visual analog scale (VAS), where 0 represented the absence of pain, and 10 represented the highest level of pain. Cancer antigen (CA) 125 serum levels were measured the day before surgery.

**Figure 1:**
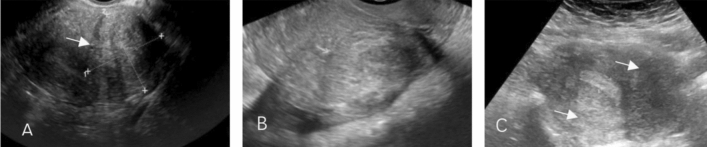
Two-dimensional transvaginal ultrasound images of adenomyosis. (**A**) with anterior adenomyosis; (**B**) with posterior adenomyosis; (**C**) with both anterior and posterior adenomyosis.

Surgical data were also collected from the laparoscopic files. Each patient’s revised American Society for Reproductive Medicine (rASRM)^9^ classification based on the revised American Fertility Society (rAFS) Score, and EFI (Endometriosis Fertility Index) score^10^ were collected according to the patients’ surgical records and infertility history. Post-surgical symptoms and pregnancy outcomes were collected from follow-up interviews with the patients. Adenomyosis diagnosis^1^ was established through a combination of physical examination, imaging, and pathology. A physical examination and two-dimensional color Dopper transvaginal ultrasound (2D-TVS) (Fig. 1) were performed the day before surgery for clinical diagnosis, and the diagnosis was further verified by histology (adenomyosis resection or biopsy) or MRI. For the purposes of the present study, the patients were divided into three groups according to the location of adenomyosis (anterior, posterior, both posterior and anterior) identified by ultrasound.

### Ultrasound diagnostic criteria

The MUSA (International Morphological Uterus Sonographic Assessment) summarizes the features of adenomyosis as^11^ an enlarged globular uterus, an asymmetrical thickening of the myometrium, myometrial cysts, an echogenic subendometrial lining and buds, hyperechogenic islands, fan-shaped shadowing, an irregular or interrupted junctional zone, and translesional vascularity on a color Doppler ultrasound examination. A thickened myometrial junctional zone^12^ was also included in the diagnosis criteria.

### Statistical analysis

Continuous data that didn’t conform to the normal distribution were presented as the median (range) and were compared using the non-parametric Kruskall–Wallis test. Continuous data that conforms to the normal distribution were presented as mean ± Standard deviation (SD) and compared using Analysis of Variance (ANOVA). Categorical data are described based on the number of patients (including percentages) and were compared using Fisher’s exact test or the chi-square test. Post-hoc test was used for interpretation differences between groups. The Kaplan–Meier method was used to calculate the cumulative probability of pregnancy. All of the analyses used a two-tailed α of 0.05 and were performed using SPSS software (Version 20.0, IBM Corp., Armonk, NY, USA). *P* < 0.05 was considered to be statistically significant.

### Ethics approval and consent to participate

This retrospective observational study was approved by the PUMCH Institutional Review Board (No. S-K1055). Signed informed written consent was obtained from all of the included patients.

## Results

### Patient characteristics

In this study, 32 (20.3%) patients had lesions on the anterior side (Group A), while 70 (44.3%) patients had lesions on the posterior side (Group B). There were 56 (35.4%) patients (Group C) who had adenomyosis on both the anterior and posterior sides. The patient characteristics for all of the groups are shown in Table 1. There were no significant differences between group A, B, and C in terms of age, BMI, gravidity, CA125, previous surgeries (endometriosis or uterus), and uterus size (*p* > 0.05). However, a significant higher parity was found in Group C compared with other groups (*p* = 0.001). There was also a significant difference among the three groups in terms of previous miscarriages. The proportion of patients with a history of previous miscarriage was significantly higher in Group C than in Group B (*P* = 0.036).

**Table 1:** Patient characteristics according to adenomyosis location.

	Group A (N = 32)	Group B (N = 70)	Group C (N = 56)	*P* value
Age (years)	34.28 ± 4.2	34.61 ± 4.0	33.86 ± 5.3	0.94
BMI (kg/m^2^)	21.80 ± 2.9	22.22 ± 3.3	22.30 ± 3.2	0.19
Gravida				0.25
0	15 (46.9%)	40 (57.1%)	26 (46.4%)	
1	10 (31.3%)	19 (27.1%)	12 (21.4%)	
≥ 2	7 (21.9%)	11 (15.8%)	18 (32.2%)	
Parity				0.001
0	28 (87.5%)_a_	61 (87.1%)_a_	33 (58.9%)_b_	
1	4 (12.5%) _a,b_	9 (12.9%)_b_	20 (35.7%)_a_	
2	0	0	3 (5.4%)	
CA125 (U/ml)	64.96 (16.1–250.2)	97.93 (8.6–958)	117.27 (25.8–661.8)	0.96
History of miscarriage	5 (15.6%)_a,b_	10 (14.3%)_b_	18 (32.1%)_a_	0.036
History of endometriosis surgery	4 (12.5%)	7 (10.0%)	7 (12.5%)	0.89
History of uterine surgery	13 (40.6%)	28 (40.0%)	23 (41.1%)	0.99
Mean uterus diameter (cm)	5.3 (3.9–9.5)	5.2 (3.4–11.5)	5.2 (3.9–11.2)	0.68
Max uterus diameter(cm)	5.85 (4.4–11)	5.80 (3.5–12.1)	5.75 (4.3–11.8)	0.99

Data are presented as mean ± standard deviation, counts (percent), or median (range) as appropriate.

*BMI* body mass index, *CA 125* carbohydrate antigen 125.

Each subscript letter indicates a subset of group categories whose proportions are not significantly different from each other (p ≥ 0.05).

### Clinical symptoms

The clinical symptoms according to the location of adenomyosis were presented in Table 2. Menstrual characteristics including length of menstrual cycle, length of period showed no statistically difference between the three groups (*p* > 0.05). A higher rate of HMB (heavy menstrual bleeding) was found in Group B (28.6%) than Group C (12.5%) (*p* = 0.04). All three groups showed no significant differences in pain symptoms, VAS score, rate of rectal tenesmus and diarrhea or constipation as well as dyspareunia, and CPP rate (*p* > 0.05).

**Table 2:** Relationship between the clinical symptoms and adenomyosis location.

	Group A (N = 32)	Group B (N = 70)	Group C (N = 56)	*P* value
Length of menstrual cycle(day)	27.75 (23–35)	28.56 (15–60)	29.24 (23–43)	0.26
Length of period(day)	5.91 (3–10)	6.12 (3–15)	6.09 (2–15)	0.74
Duration of dysmenorrhea(month)	60 (0–180)	54 (0–288)	48 (0–360)	0.45
Pain symptoms, n(%)	21 (65.6%)	54 (77.1%)	45 (80.4%)	0.29
Dysmenorrhea (VAS)	3 (0–10)	6 (0–10)	6.5 (0–10)	0.06
Dyspareunia, n(%)	2 (6.3%)	8 (11.4%)	8 (14.3%)	0.52
Rectal tenesmus, n(%)	3 (9.4%)	19 (27.1%)	13 (23.2%)	0.12
Intestinal symptoms, n(%)	2 (6.3%)	14 (20.0%)	5 (8.9%)	0.07
CPP, n(%)	3 (9.4%)	9 (12.9%)	10 (17.9%)	0.50
HMB, n(%)	4 (12.5%)_a,b_	20 (28.6%)_b_	7 (12.5%)_a_	0.04

Data are presented as mean ± standard deviation, counts (percent), or median (range) as appropriate.

*CPP* chronic pelvic pain, *VAS* visual analogic scale, *HMB* heavy menstrual bleeding.

Each subscript letter indicates a subset of group categories whose proportions are not significantly different from each other (p ≥ 0.05).

### Surgical findings

The three groups had no statistical differences in terms of coexistence of deep infiltrating endometriosis. However, the combination of peritoneal endometriosis and ovarian endometrioma was significantly different among three groups. Group C had a significantly lower rate of concurrent peritoneal endometriosis compared to Group A and Group B (*P* = 0.01). Higher concurrence rate of ovarian endometrioma was also observed in Group C (*p* = 0.002) (Table 3). Concerning the combination of other gynecologic diseases, Group B showed a relative higher rate of coexistent oviduct obstruction (24.3%, *P* = 0.038). Group A had a higher proportion of coexistent leiomyoma (53.1%, *P* = 0.002). Group C presented a higher rate of coexistent pelvic adhesion (80.4%, *P* = 0.003) and exhibited a higher r-AFS score than Group A and Group B (*P* < 0.001). There were no significant differences between group A, B, and C in terms of endometrial polyps, operation time, and EFI score (*p* > 0.05).

**Table 3:** Surgical findings and adenomyosis location.

	Group A (N = 32)	Group B (N = 70)	Group C (N = 56)	*P* value
Endometriosis status				
SPE	15 (46.9%)_a_	28 (40.0%)_a_	11 (19.6%)_b_	0.01
OEM	14 (43.8%)_a_	44 (62.9%)_a,b_	45 (80.4%)_b_	0.002
DIE	13 (40.6%)	23 (47.1%)	24 (42.9%)	0.81
AM type				< 0.001
Diffuse	12 (37.5%)_a_	32 (45.7%)_a_	53 (100%)_b_	
Focal	20 (62.5%)_a_	38 (54.3%)_a_	0 (0%)_b_	
Leiomyoma	17 (53.1%)_a_	26 (37.1%)_a,b_	10 (17.9%)_b_	0.002
Oviduct obstruction	6 (18.8%)_a,b_	17 (24.3%)_b_	4 (7.1%)_a_	0.038
Endometrial polyps	8 (25.0%)	18 (25.7%)	6 (10.7%)	0.09
Pelvic adhesion	15 (46.9%)_a_	52 (74.3%)_b_	45 (80.4%)_b_	0.003
Operation time (min)	60 (30–120)	60 (20–180)	80 (20–300)	0.14
EFI	7 (5.5–8.5)	6 (4–8)	5.5 (5–8)	0.66
rAFS	22 (2–116)_a_	48 (1–112)_b_	64 (2–100)_c_	< 0.001

Data are presented as mean ± standard deviation, counts (percent), or median (interquartile range) as appropriate.

*SPE* superficial peritoneal endometriosis, *OEM* ovarian endometrioma, *DIE* deep infiltrating endometriosis, *EFI* endometriosis fertility index, *rAFS* revised American Fertility Society.

Each subscript letter indicates a subset of group categories whose proportions are not significantly different from each other (p ≥ 0.05).

### Pregnancy outcomes

**Figure 2:**
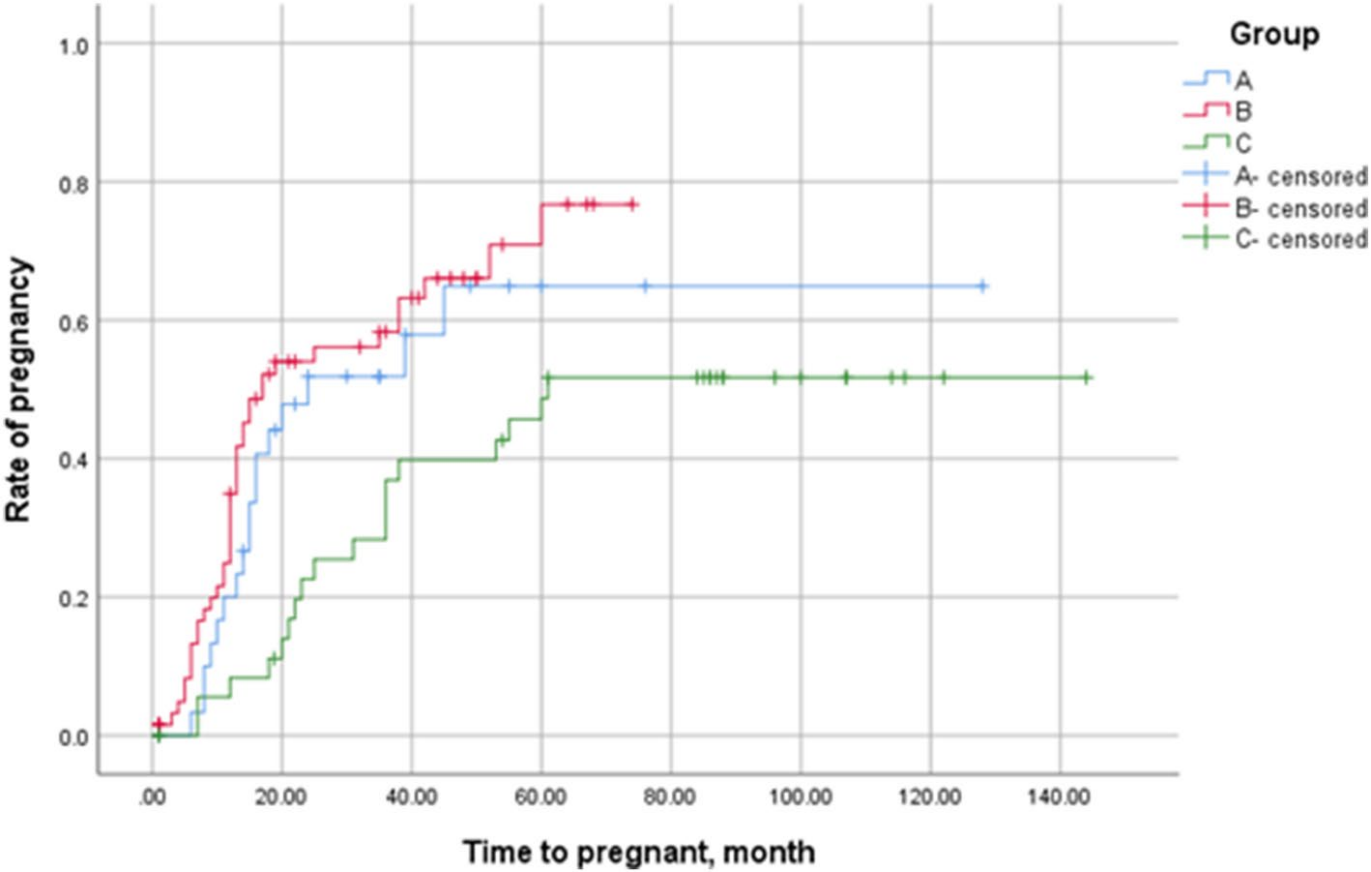
The overall pregnancy rate during the long-term follow-up. Kaplan–Meier curves presenting the cumulative pregnancy rate according to the time after conservative surgery. There were significant differences observed among the three groups according to the log-rank test analysis (χ^2^ = 9.189, *p* = 0.01).

A total of 133 patient were followed up (median 50 months, range 1–144 months). Among them, 103 patients provided details of their post-operative pregnancy during follow-up, and 61 (59.2%) experienced a clinical pregnancy. A total of 16(26.2%) patients suffered pregnancy loss, with 14 patients experiencing pregnancy loss before 12 weeks and 2 experiencing pregnancy loss after 12 weeks. A total of 65(63.1%) patients underwent IVF-ET. The total IVF pregnancy rate was 64.6%(42/65) and total spontaneous pregnancy rate was 50.0%(19/38) (*p* = 0.145). 45 patients (33 CS (cesarean section) vs. 12 VD (vaginal delivery)) patients took their baby home. The detailed pregnancy and obstetrical outcomes of the three groups were showed in Supplementary Table 1. As for obstetric complications, even though no significant differences were observed, we found that placenta previa, placenta accreta, preeclampsia, and preterm birth were mainly observed in Group B and Group C (with lesions located in the posterior side). Survival analysis using the Kaplan–Meier test demonstrated significant differences (*p* = 0.01) in the cumulative pregnancy rate among the three groups (Fig. 2).

## Discussion

In our population of patients who underwent surgery for benign gynecologic diseases, we observed different clinical profiles as well as pregnancy outcomes depending on the location of adenomyosis. First, we analyzed the distribution of adenomyosis and found that most (44.3%) patients had lesions mainly on the posterior wall. This was consistent with Exacoustos’s study, which evaluated the ultrasound features of 43 adolescents and found that the posterior uterine wall (58%) and the outer myometrial layer (93%) were the most affected areas^13^. In our study, history of delivery and miscarriage was found more often in patients with adenomyosis on both anterior and posterior wall, suggesting a possible correlationship between pregnancy and this subtype of adenomyosis. The association of parity and adenomyosis was supported by several studies^14–17^. A possible explanation^18^ might be the trophoblast invasion of the inner myometrium during pregnancy that disrupt the junctional zone (JZ). However, adenomyosis was also found to be related with infertility and this theory couldn’t explain those patients with primary infertilty.

Regarding the clinical features, our study found that the anterior group tended to have the lower VAS scores, lower rate of pain symptoms (dyspareunia,rectal tenesmus, intestinal symptoms and CPP), although there were no significant differences. The P value was just slightly above 0.05 in VAS score (*p* = 0.06) and Intestinal symptoms (*p* = 0.07). There might be statistical differences if we could have larger the sample size in the future. Our data also denoted that there were significant differences between groups in heavy menstrual bleeding (HMB) (28.6% in group B compared with 12.5% in group A and C seperately). Many studies have investigated clinical profiles according to the adenomyosis phenotype. They have tried to summarize how the features that have been identified can be linked to specific clinical manifestations and could thus help make correct treatment decisions. Earlier research focused on histological features. In a previous study that included histopathologic slides obtained from 94 women with adenomyosis, there was a significant correlation between the depth of penetration and the number of adenomyosis foci (r = 0.3446; *p* = 0.0001). However, the symptoms did not correlate with the degree of penetration^19^. In another study that analyzed six groups of women with adenomyosis (anterior and posterior cuts on the uterus at the cervix, lower uterine segment, and fundus), Blanco et al.^20^ showed that were significant differences that could be observed in terms of the number of nests at all levels (*p* < 0.001), but no statistical differences between the anterior and posterior regions of each level could be determined when only evaluating for the presence of disease. It is gradually being recognized that symptoms may not correlate with the depth of invasion or the extent of disease^4^. Previous studies showed some differences in clinical symptoms between focal and diffuse adenomyosis. Uyar et al.^21^ retrospectively analyzed 755 hysterectomy cases in which adenomyosis was diagnosed. They found that endometrial diseases were more common in diffuse adenomyosis as were asymptomatic and incidental adenomyosis. While abnormal uterine bleeding (AUB) was more frequently associated with nodular adenomyosis. In a multicenter, observational, prospective study reported by Exacoustos et al.^22^, women with diffuse adenomyosis were older and experienced heavier menstrual bleeding compared to those with focal disease, but there were no statistically significant differences in the severity of dyspareunia and dysmenorrhea. It should be noted that adenomyosis is much more complicated and challenging. No certain correlations between disease classification and specific clinical symptoms have been observed.

With the development of imaging technique, adenomyosis is mostly diagnosed by non-invasive methods such as US or MRI recently. Kishi et al.^23^ initially defined four adenomyosis subtypes according to MRI-based diagnosis: intrinsic, extrinsic, intramural, and indeterminate adenomyosis. In their study, patients with the diffuse internal adenomyosis subtype were older (38.7 years vs. 36.9 years, *p* < 0.05) and had a more frequent history of uterine curettage (32.2% vs. 7.8%, *p* < 0.01), while those with focal adenomyosis of the external myometrium subtype were more often nulligravid (35.3% vs. 57.6%, p < 0.05), and the latter subtype was more commonly combined with EM in the posterior cul-de-sac (92.3% vs. 25.4%). Chapron et al.^24^. later defined two adenomyosis subtypes: diffuse internal adenomyosis and focal adenomyosis of the external myometrium. According to their study^5^, women with external adenomyosis were significantly younger (31.9 ± 4.6 vs. 33.8 ± 5.2 years; *P* = 0.006), more often nulligravid (*P* < 0.001), and more likely to be associated with DIE (*P* < 0.001). These findings were further supported by histological findings, as differences in biomarker expression were used to link DIE to extrinsic adenomyosis^25^. While internal adenomyosis was more often associated with HMB, no differences in the pain scores were observed between the two groups. A new classification proposal^7^ published in 2020 suggests we should classify adenomyosis based on five main categories that have been adapted from the original concept: affected area, pattern, size (volume), the localization of adenomyotic lesions, and concomitant pathologies. Here, localization was defined as anterior, posterior, left lateral, right lateral, or fundal. Marcellin, L. et al. tried to evaluate the association between deep infiltrating endometriosis (DIE) in the bladder and anterior focal adenomyosis of the outer myometrium (aFAOM); however, the link remains unclear^26^. Based on the limited amount of research that is available, the impact of location on the severity of the disease's clinical presentation is still unclear. It is noteworthy to underline that based on our results, the anatomical location could potentially be related to HMB.

Adenomyosis is associated with many pregnancy complications^27,28^, including but not limited to infertility, early pregnancy loss, growth restrictions, preterm delivery, and preeclampsia. They might affect both the mother and fetus with possible long-term sequelae. According to our data, 16(26.2%) of the patients experienced pregnancy loss, which was consistent with the meta-analysis^29^ published by Vercellini et al. in 2014 (miscarriage rate of 31% in AM). A prospective randomized study^30^ reported that the presence of the adenomyosis within the uterus was found to be more common in patients with preeclampsia and fetal growth restrictions compared to patients without fetal growth restrictions (94.4 vs. 64.7%; *p* = 0.041). Indirect signs of AM on an MRI might be associated with late-onset preeclampsia (*p* < 0.05). Currently, infertility is considered to be associated with the specific location of the adenomyosis lesions, but this is not the case for all phenotypes. Focal disease was associated with a higher percentage of infertility^22^. In a cross-sectional study of 496 women^31^, a significant relationship between the presence of FAOM and primary infertility (*p* < 0.01) was shown, while diffuse adenomyosis of the internal myometrium was not associated with either primary or secondary infertility. Kim et al.^32^ reported a preterm labor rate of 24.56% in a retrospective study and found that uterine wall thickness in the second trimester was related to subsequent preterm delivery in pregnancies with adenomyosis. Our previous study also demonstrated that the size of the uterus was significantly smaller in those who had a successful delivery compared to those who did not have a live birth (*p* = 0.001)^33^. So far as we know, little research has reported the differences among pregnancy outcomes based on whether AM is located at the anterior, posterior, or both sites. Our survival analysis demonstrated a significant lower cumulative pregnancy rate in the both sides group than other groups. The reason might be a wider invasion of adenmyosis for both sides group. Besides, Group C also had a higher concurrence rate of OEM (ovarian endometrioma), pelvic adhesion and rAFS score, which might affect pregnancy rate. We found that severe obstetric complications such as placenta previa, placenta accreta, preeclampsia, and preterm birth were only present in women who had adenomyosis lesions at the posterior side (either only at the posterior side or both at anterior and posterior sides). Overall, the various potential complications associated with adenomyosis in pregnancy could be related to the anatomical location of adenomyosis. The pathophysiologic mechanisms of these complications and their relationship to adenomyosis during pregnancy are not fully understood. Zhang et al.^34^ investigated 95 pregnant patients with adenomyosis and found that patients with pregnancies complicated by adenomyosis are prone to adverse pregnancy outcomes (placental abnormalities, fetal distress, preterm delivery, intrapartum bleeding, gestational weeks, and neonatal birth weight) if embryo implantation is located on or very close to the adenomyotic lesions (all *p* < 0.05). An activation of the inflammatory pathways and defective myometrial spiral artery remodeling were considered to be the major causes^28^. Further research on molecular mechanisms is needed.

However, our study also has several limitations. First, AM is also frequently associated with other gynecologic diseases, including fibroids^35^ and endometriosis^36^. It was reported that EM was prevalent in 21.8–80.6% of patients with AM and that AM concomitantly existed in 79–91.1% of patients with EM^37–39^. According to Chapron et al.^5^, endometriosis is found in 96.3% of patients presenting with adenomyosis of the external myometrium. In our study, Group C demonstrated a higher combination rate of OEM, pelvic adhesion and rAFS score compared the anterior group. There might be a correaltionship between the extent of adenomyosis and the severity endometriosis. N. Berlanda et al.^40^ reported an increased risk of placenta previa and cesarean delivery when severe adenomyosis is coexistent with endometriosis. A significant correlation with pregnancy-induced hypertension and preeclampsia was reported in another study^41^ in Italy. We also found a total of 53(33.5%) patients combined with leiomyoma, with almost half of the patients in group A combined with leiomyoma. A total of 32(20.3%) patients combined with endometrial polys with no signifcant differences among three groups. Therefore, adenomyosis is a difficult disease to study in isolation^42^, and we couldn’t exclude those combined benign gynecologic diseases during the Real-World Study. Second, we did not include patients who were older than 40 years old. Increased age, a risk factor for adenomyosis^43^, is also one of the most important factors affecting infertility and obstetric complications. Future studies could include patients older than 40 years of age. Environmental factors^44,45^ were also reported to be linked to reproductive dysfunction. Third, surgeries might impact the pregnancy outcome, however we could not just perform diagnostic sugery. In addition, the study population was selected from one referential center for diagnosis. A multiple center clinical trial with a larger sample size is needed.

## Conclusions

Our findings suggest the location of adenomyosis in the myometrium based on sonography appeared to have different impacts on clinical symptoms as well as pregnancy outcomes. Patients with adenomyosis lesion in both anterior and posterior lesions had higher concurrence rate of OEM, pelvic adhesion and rAFS score while lower cumulative pregnancy rate. Severe obstetric complications were only observed groups when there were lesions in posterior sides. Accurate evaluation of adenomyosis lesions in the posterior uterus wall with ultrasound, followed by closer fetal monitoring, and referral to tertiary care might be helpful in decreasing obstetrical complications. We believe that our results will be helpful for making treatment plans. However, additional research based on larger study populations is necessary to confirm these findings.

### Supplementary Information

Supplementary Information.

## Data Availability

Correspondence and requests for materials should be addressed to Prof. Jinhua Leng.
