# Supplementary material for: Effects of localization of uterine adenomyosis on clinical features and pregnancy outcome
Source: Sci Rep. 2023 Sep 7;13:14714. doi: 10.1038/s41598-023-40816-z (PMC10485030; doi:10.1038/s41598-023-40816-z)
Supplement: Supplementary file 1 — Supplementary Information. [file 41598_2023_40816_MOESM1_ESM.docx]

Supplementary Table I. Pregnancy outcome according to adenomyosis location.

|  | GroupA(N=22) | Group B(N=52) | Group C(N=29) | P-value |
| --- | --- | --- | --- | --- |
| Clinical pregnancy rate n (%) | 15（68.2%） | 33（63.5%） | 13（44.8%） | 0.165 |
| Pregnancy loss | 5(33.3%) | 9(27.3%) | 2(15.4%) | 0.549 |
| Pregnancy loss before 12 weeks /clinical pregnancy, n (%) | 4(26.7%) | 8(24.2%) | 2(15.4%) | 1.0 |
| Pregnancy loss after 12 weeks /clinical pregnancy, n (%) | 1(6.7%) | 1(3.0%) | 0(0%) |  |
| Live birth rate, n (%)  IVF  Natural | 10(45.5%)  6(60%)  4(40%) | 24(46.2%)  11(45.8%)  13(54.2%) | 11（37.9%）  8(72.7%)  3(27.3%) | 0.76  0.35 |
| malformation | 1(4.5%) | 0 | 0 | 0.478 |
| Ectopic pregnancy | 1(4.5%) | 0 | 1（3.4%） | 0.222 |
| GDM/live birth, n (%) | 3(30.0%) | 6(25.0%) | 3（37.3%） | 1.0 |
| PROM/live birth, n (%) | 2(20.0%) | 1(4.2%) | 2(18.2%) | 0.233 |
| CS/live birth, n (%)  Twin  Abnormal fetal position  fetal distress  failure in induction of labor  operation history  fetal macrosomia  placenta previa  Heartfailure | 6(60.0%)  0  1  0  1  3  1  0  0 | 19(79.2%)  6  2  0  0  2  4  4  1 | 8（72.7%）  3  1  0  0  2  0  2  0 | 0.493 |
| placenta accreta/live birth, n (%) | 0 | 1（4.2%） | 1（9.1%） | 0.721 |
| preeclampsia/live birth, n (%) | 0 | 2（8.3%） | 1（9.1%） | 1.00 |
| Preterm birth/live birth, n (%) | 0 | 5（20.8%） | 3（27.3%） | 0.291 |

### Data are presented as n (%) as appropriate.

### Abbreviations: IVF, in vitro fertilization; GDM, gestational diabetes mellitus; PROM, premature rupture of membranes; CS, [cesarean section](https://www.baidu.com/link?url=ALF7ibh3CerqCXyzRTwZK-jSY_0UU4BFOlfHvd8H4Rf06zSEeFkYD60TWg2CFeNX&wd=&eqid=972af67b000829470000000261dbcf38).
